# Immune Response of Elite Enduro Racers to Laboratory and Racing Environments: The Influence of Training Impulse and Vibration

**DOI:** 10.3390/ijerph18094603

**Published:** 2021-04-26

**Authors:** Lewis Kirkwood, Lesley Ingram-Sills, Mark Dunlop Taylor, Eva Malone, Geraint Florida-James

**Affiliations:** 1 School of Applied Sciences, Edinburgh Napier University, Edinburgh EH11 4BN, UK; L.Ingram-Sills@Napier.ac.uk (L.I.-S.); E.Malone@Napier.ac.uk (E.M.); G.Florida-James@Napier.ac.uk (G.F.-J.); 2 Mountain Bike Centre of Scotland, Peel Tower, Glentress EH45 8NB, UK; 3 School of Engineering and the Built Environment, Edinburgh Napier University, Edinburgh EH10 5DT, UK; M.Taylor@Napier.ac.uk

**Keywords:** mountain biking, training load, recovery, leukocyte redistribution

## Abstract

Introduction: Understanding the sport-specific immune response elicited during both training and competition is imperative to maximise athlete health and performance. Despite a growing population of professional enduro mountain bike athletes, little is known about the recovery of the immune system following enduro racing events. Methods: Nine international level elite enduro mountain bike athletes (age 24.3 ± 2.4 years, height 178.5 ± 8.7 cm, mass 76.5 ± 12.5 kg) completed a laboratory-based maximal exercise test (LAB) on a cycle ergometer and competed in an international mountain bike enduro race event (RACE). Blood samples were taken before, immediately after, and 1 h after LAB and before, 1 h after, and 17 h after RACE. Leukocyte subsets were enumerated using seven-colour flow cytometry. Lucia’s training impulse (LuTRIMP) and vibration exposure (VIB) were quantified during RACE. Results: Seven participants were included in the final analyses. There was a significant (*p* < 0.05) increase in neutrophil count alongside a reduction of cytotoxic lymphocyte cell subsets of both the innate (CD3^−^/CD56^+^ NK-cells and CD3^−^/CD56^dim^ NK-cells) and adaptive (CD8^+^/CD62L^−^/CD45RA^−^ T-cells and CD8^+^/CD27^+^/CD28^−^ T-cells) components of the immune system one hour after RACE. All cell counts returned to baseline values 17 h afterwards (*p* > 0.05). Cell subset redistribution from pre- to post-one-hour time points (%Δpre-post1h) in cell subsets with potent effector functions (Neutrophils, CD3^−^/CD56^+^ NK-cells, CD8^+^/CD62L^−^/CD45RA^−^ T-cells, CD8^+^/CD27^+^/CD28^−^ T-cells, and CD3^−^/CD56^dim^/CD57^−^ NK-cells) was significantly greater at RACE than LAB (*p* < 0.05). VIB was shown to be a superior predictor of %Δpre-post1h CD4^+^ T-cells, CD4^+^ early T-cells, CD4^+^ naïve T-cells, and NK cells as compared with LuTRIMP on its own (Δ*R*^2^ = 0.63 − 0.89, *p* < 0.05). Conclusions: The race event offers a greater challenge to the immune system than LAB, and potentially, whole body vibration is a key component of training load measurement in mountain bike applications.

## 1. Introduction

Days of training missed due to illness or injury can limit training volume for endurance athletes [1] which can then have a detrimental effect on performance [2]. However, upper respiratory tract and pulmonary infection risk has been shown to increase 8–10% with each 10% increase in training load and by 50–70% during periods of intensified training in swimmers [3]. Similarly, periods of intensified training in endurance-trained cyclists have shown reductions in CD8 and CD4 T-cell redistribution in response to exercise [4]. Indeed, while transient immune disturbances induced by acute exercise may be beneficial for immune function under provision of adequate recovery [5,6], sustained periods of insufficient recovery may lead to chronic immune suppression [7]. Interestingly, a recent study in mice showed that the mechanical loading associated with exercise led to the secretion of osteogenic growth factor by mesenchymal stromal cells in the bone marrow, in turn signaling an enhanced defensive immune response to threaten infection by increasing lymphoid progenitors and increased lymphocyte differentiation [8].

Immediately post laboratory exercise in healthy athletes, leukocytosis magnitude is driven by an increased number of neutrophils and effector lymphocyte subsets [9,10] dependent on exercise intensity and duration relative to training status. One hour after laboratory exercise, the magnitude of reduction in effector memory lymphocyte count and increase in neutrophil count is also driven by exercise intensity and duration relative to training status [11]. Periods of excessive heavy training can reduce the expression of the β2-adrenergic receptor, in turn blunting the mobilisation of NK cells and cytotoxic T-cells in response to exercise [12,13]. Concentrations of cytokines and glucocorticoids are also often altered during exercise and subsequent recovery period, depending on exercise intensity and duration relative to training status [14,15]. However, psychological stress [16] and environmental factors [17] associated with outdoor races may substantially influence the immune response, suggesting field data are necessary to understand this fully. Previous field data have shown that peripheral blood lymphocyte count increased, decreased, or remained unchanged after competitive half or full marathon running [18,19,20,21,22], although this inconsistency may be due to difficulties in blood sampling immediately upon exercise cessation in a competitive environment [23]. Peripheral blood lymphocyte counts returned to baseline levels between 16 and 24 h later [21,24], proposed to be of benefit to the cellular immunity of the athletes in these studies.

While previous studies have focused on competitive running events, little is known regarding the immune response to the rapidly expanding sport of enduro mountain bike racing. Enduro mountain bike racing consists of timed downhill race stages linked by noncompetitive transition stages and a general classification is determined by accumulated race stage time. Enduro events typically cover a wide range of challenging, rough terrain including roots, rocks, and jumps [25,26]. Elite enduro mountain bike athletes train and compete on successive days on a regular basis, highlighting the requirement to investigate the recovery of the immune system following competition. The demands of enduro mountain bike racing are unique among cycling disciplines, placing substantial aerobic and anaerobic workload demands on the athletes over prolonged durations in conjunction with the requirement to attenuate large terrain-induced vibration [25,26]. Considering that acute vibration exposure can induce transient changes in CD4 and CD8 T-cell count [27], it is possible that terrain-induced vibration may influence the immune response to enduro mountain bike competition. Therefore, given that training load must be prescribed alongside sufficient rest periods to allow the immune system to recover from subsequent bouts [28], the response of the immune system after an enduro mountain bike competition is worthy of further investigation. Therefore, the aims of this study were the following: (1) To detail the redistribution of leukocyte subsets in response to laboratory-based maximal exercise and field-based international enduro mountain bike racing, and (2) to investigate the relative contribution of training impulse and vibration on cell subset redistribution one hour after an international enduro mountain bike race.

## 2. Materials and Methods

### 2.1. Participants

Eight male (age = 25 ± 3 years, height = 181 ± 6 cm, mass = 80 ± 11 kg) and one female (age = 23, height = 160 cm, mass = 56.6 kg) elite enduro mountain bike athletes (mean best Enduro World Series (EWS) finish = 30 ± 35, training history 10 ± 4 years, average weekly training duration = 11 ± 3 h/week) agreed to participate in this study. The study was conducted according to the guidelines of the Declaration of Helsinki and ethical approval for the study was granted from the ethics committee of Edinburgh Napier University. Subsequently, informed consent was obtained from all subjects involved in the study. Participants were free from any perceived illness for two weeks prior to all testing, were not using medication, and were required to refrain from consuming alcohol or caffeine in the 24 h preceding the laboratory visit and race event. Participants were instructed to fast for 12 h prior to the laboratory test. Diet was not controlled for the race event to minimize interference with the athlete’s performance in a high-profile live racing event. Participants were also requested to abstain from training for 24 h prior to both the laboratory test and the resting blood sample for the race event. As course practice is mandatory in the days following the resting blood sample and immediately preceding the race event, it was not possible to control training load on these days.

### 2.2. Laboratory Exercise Protocol

The exercise protocol began between 10.30 a.m. and 11 a.m. to minimise the influence of circadian variation [29]. Heart rate at fixed blood lactate concentrations of 2 and 4 mmol·L^−1^ (FBLC2 and FBLC4, respectively) was assessed using an incremental exercise test on a bicycle suited to the height of the participant (Surprise, size large, Pinarello, Italy; Alpha 1.2, size 56 cm, Trek, Taiwan; Mira, size small, Litespeed, Ooltewah, TN, USA) mounted on a cycle ergometer (Computrainer Pro 3D, RacerMate, Seattle, WA, USA). Blood samples, taken from the ear lobe, were analysed for blood lactate concentration using the Lactate Pro 2 Meter (Lactate Pro2 LT-1730, Arkray, Kyoto, Japan), which has been previously reported to be a reliable (CV ≤ 1.0%) measure of blood lactate concentration [30]. The initial workload was set at 110 W increasing 30 W every 3 min. Samples were taken within the last 30 s of each workload until lactate concentration was ≥4 mmol·L^−1^. At this point the workload increased by 20 W every minute until volitional exhaustion or when cadence >60 rpm could not be maintained.

### 2.3. Race Event Protocol

**Figure 1:**
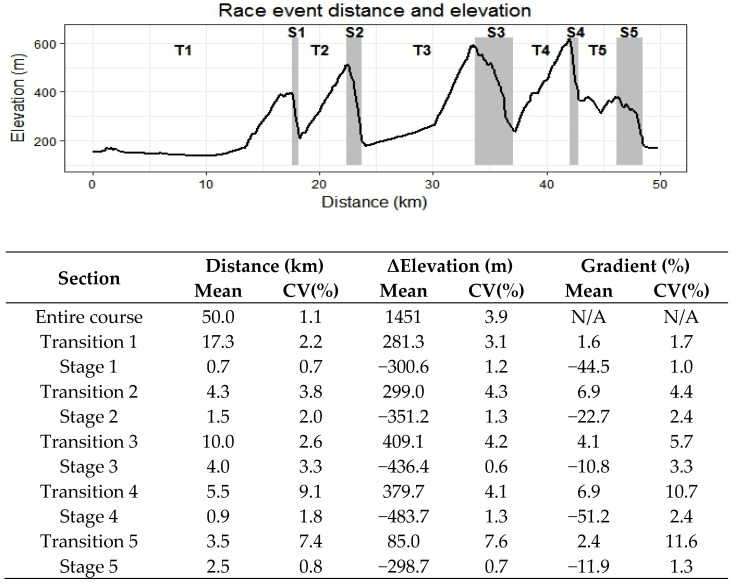
Distance, elevation, and gradient details for the entire course, stage (S), and transition (T) sections (all data are presented as mean and coefficient of variation (CV).

A one-day international enduro race in the United Kingdom consisting of five race stages was chosen for the race analysis. The race stages featured a range of technical terrain as previously described [25,26]. Weather conditions were 10 °C, overcast, and the tracks were damp. Mean distance, elevation, and percentage gradient ($100\times \frac{\Delta elevation\;\left(m\right)}{distance\;\left(m\right)}$) of the race stages, transition stages, and entire course are available in Figure 1. These data were not provided by organisers and were instead calculated from the global positioning system (GPS) device of each athlete, hence, the coefficient of variation (CV) was also provided as a measure of reliability. Participants rode their own bikes for the race, all of which were typical full suspension enduro bikes with 140–170 mm of travel front and rear.

### 2.4. Heart Rate and Location Data

Location and elevation were measured at 10 Hz using both GPS and global navigation satellite system (GLONASS, Catapult Optimeye S5, Catapult Innovations, Melbourne, Australia). All participants wore a wireless HR monitor strap (Wearlink, Polar, Finland) encoded to their individual GPS units recording HR at 1 Hz. Due to the limited battery life of the GPS units and the long first transition, participants were instructed to turn the units on 10 min prior to the start of the first stage. GPS data (10 Hz sample rate) was used to calculate overall run time (s) in agreement with event organisers’ time to allow calculation of heart rate and partial vibration exposure data for each race stage. The distance and elevation of the first transition stage was calculated using data from the participants’ own GPS cycling computers (Garmin Edge 500, Garmin, Lenexa, KS, USA). Upon return to the event headquarters, the Catapult device unit was connected to a laptop via a USB connection and the data were transferred for post-processing.

### 2.5. Training Load

Lucia’s training impulse (LuTRIMP) model was used to calculate the internal training load for the overall race event using FBLC2 and FBLC4 HR values calculated during laboratory testing (see Section 2.2 above). Time spent at HR < FBLC2 was provided a weighting factor of 1, time at HR between FBLC2 and FBLC4 a weighting factor of 2, and time at HR > FBLC4 a weighting factor of 3. Time spent in each heart rate zone was multiplied by the relevant weighting factor and summated to form Lucia’s TRIMP [31,32].

### 2.6. Seat Post Vibration Measurement

An inertial measurement unit (IMU) featuring a 100 Hz triaxial accelerometer (Catapult Optimeye S5, Catapult Innovations, Melbourne, Australia) was fixed to the seat post of each participant’s bicycle. This location was selected to minimise ergonomic interference which could hinder the athlete’s performance or reduce safety. The Catapult device external dimensions were 96.5 × 52 × 14 mm³ and the device weighed 0.067 kg. The accelerometer was orientated with the *x*-axis vertically in line with the seat post tube, the *y*-axis directed to the right-hand side of the rider, and the *z*-axis rear facing. Sensitivity was set at ±16 g. Seat post vibration measurements were calculated for each stage as root mean square of the combined three axes accelerations (rms, ms^−2^). The rms acceleration value was calculated using:

(1) $$rms=\sqrt{a_{hwx}^{2}+a_{hwy}^{2}+a_{hwz}^{2}}$$

Adapting the methodology outlined in the ISO guidelines for whole body vibration [33], partial saddle vibration exposure values were combined to calculate total saddle vibration exposure for all race stages using the following:

(2) $$Vibration_{race}=\sqrt{\overset{n}{\underset{i=1}{\sum }}r.m.s.^{2}}$$

The accelerometer was used to provide a quantitative indication of vibration exposure for each race stage. Therefore, the measurements used were an indication of the vibration experienced by the bicycle frame at a common point.

### 2.7. Blood Sampling

**Figure 2:**
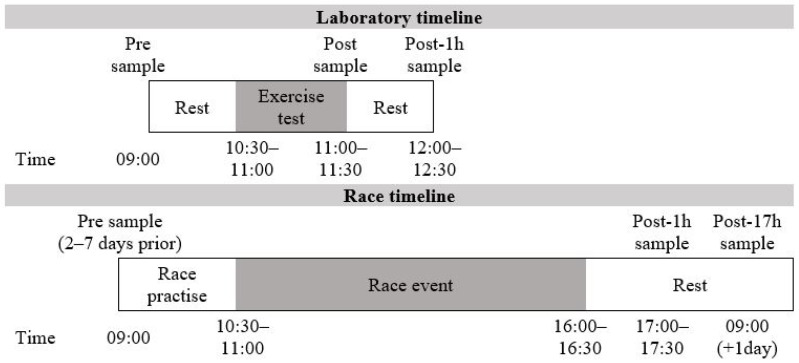
Timeline of blood sampling time points for the laboratory exercise protocol and race event.

For the laboratory exercise tests, intravenous blood was collected from participants in the supine position before (pre), immediately after (post) and 1 h after exercise (post-1h). To facilitate the collection of blood samples at the race event, a mobile laboratory was created 200 m from the finish line of the international enduro race event in Peebles, Scotland. To minimise the demands placed on the participants (elite athletes) in a racing situation, resting intravenous blood samples (pre) were collected at 9 a.m. between 2 and 7 days prior to the event (prior to practice commencing, following 24 h of rest). Intravenous blood samples were also collected 1 h after the event (post-1h) and the morning after the event (post-17h) as shown in Figure 2. Pre- and post-17h blood samples were collected at Edinburgh Napier University human performance laboratory or a mobile laboratory set up in the Mountain Bike Centre of Scotland near Peebles. All blood was drawn into 6 mL vacutainers (Becton-Dickson, Oxford, UK) coated in EDTA.

### 2.8. Blood Sample Analysis

An automated haematology analyser (XS 1000i, Sysmex, Milton Keynes, UK) was used to determine total blood leukocyte and differential counts in duplicate within one hour of sampling in the laboratory and within two hours of sampling for the race event. Plasma volume changes were estimated [34] and used to adjust cell counts and plasma hormone levels accordingly. Serum samples were collected in 6 mL serum separating tubes (Becton-Dickson, Oxford, UK) before being centrifuged at 2000× *g* for 10 min at room temperature. Upon removal, serum samples were frozen at −80 °C for further analysis. Serum cortisol was investigated at all time points, while IL-6 was only assessed before (pre) and 1 h after (post-1h) the laboratory visit and the race event. Serum cortisol and IL-6 concentrations were measured using commercially available enzyme linked immunosorbent assay (ELISA) kits (R&D Systems, Abingdon, UK), performed in accordance with the manufacturer’s instructions. Plates were read on a plate reader (Labtech LW5000, Heathfield, UK) set to a wavelength of 450 nm for both cortisol and IL-6. The intra-assay coefficient of variation was 11.4% for IL-6 and 11.0% for cortisol.

### 2.9. Lymphocyte Phenotyping

Blood samples (3 mL) were mixed with an equal volume of 0.9% sodium chloride solution (NaCl; Baxter, UK) and the 6 mL solution was layered carefully on 3 mL of lymphoprep solution (Axis-shield, Oslo, Norway), using procedures previously described [35]. Aliquots of 0.5 × 10^6^ peripheral blood mononuclear cells (PBMCs) were incubated with a BV786-conjugated anti CD3 (SK7, IgG_1_), a V450-conjugated anti CD4 (RPA-T4, IgG_1_), a V500-C-conjugated anti CD8 (SK1, IgG_1_), a BV650-conjugated anti CD27 (L128, IgG_1_), a PE-Cy 7-conjugated anti CD28 (CD28.2, IgG_1_), a PerCP-Cy 5-conjugated anti CD45RA (HI100, IgG_2b_) a BB515-conjugated anti CD62L (SK11, IgG_2a_), and 50 µL Brilliant Stain Buffer (BD biosciences, Oxford, UK) for T-cell populations or a PE-Cy 7-conjugated anti CD56 (B159, IgG_1_) and FITC-conjugated anti CD57 (HNK-1, IgM) monoclonal antibodies (mAb) for NK cells. Anti CD3, CD28, and CD45RA mAbs were diluted 1/1 and PBMCs were incubated with 5 μL of each mAB solution for 30 min at room temperature. All monoclonal antibodies were manufactured by BD Biosciences, Oxford, UK.

### 2.10. Flow Cytometry and Gating

Cell phenotyping was completed using FACSDiva software (BD Biosciences, San Jose, CA, USA) in combination with a FACSCelesta flow cytometer (BD Biosciences, San Jose, CA, USA) using a three-laser configuration. The three argon ion lasers emit light at a fixed wavelength/power of 488 nm/20 mW (blue), 561 nm/50 mW (yellowish green), and 405 nm/50 mW (violet). An electronic gate was placed around the lymphocyte population using a plot of forward scatter against side scatter. Side scatter against BV786 fluorescence was used to determine and gate CD3^+^ (T-cell) and CD3^−^ (NK cell) populations. The expressions of CD4 and CD8 were determined on the CD3^+^ gate, and CD56 expression was determined on the CD3^−^ gate. Flow cytometry dot plots were used to identify CD3^+^ CD4^+^ and CD3^+^ CD8^+^ populations and their co-expression of CD27 and CD28 or CD45RA and CD62L (see Table 1). A total of 10,000 gated CD3^+^ (T-cell) or CD3^−^ (NK cell) events were recorded for analysis of all samples. Then, total counts of lymphocyte subsets were obtained by multiplying the population percentage values from the flow cytometer by the total lymphocyte counts from the automated haematology analyser.

### 2.11. Data Analysis

A one-way repeated measures ANOVA was used to investigate differences in total cell counts for each leukocyte subset at each time point (lab: pre, post, post-1h; race: pre, post-1h, post-17h). In the event of a significant effect of time point, Bonferroni post hoc tests were employed to identify where differences occurred. To investigate differences between test (race and laboratory) and time point (pre, post-1h), a two-way repeated measures ANOVA (test * time point) was used. If the results of the two-way repeated measures ANOVA revealed a significant interaction (test * time point), Bonferroni post hoc analysis was utilised to identify differences in test or time point. If Mauchly’s test indicated the assumption of sphericity had been violated, degrees of freedom were corrected using Greenhouse–Geisser estimates of sphericity. The effect size was calculated throughout as omega squared (ω^2^). All data are reported as mean ± SD unless otherwise stated. All ANOVA tests were performed using IBM SPSS Statistics for Macintosh, version 26 (IBM Corp., Armonk, NY, USA).

Multiple linear regression was utilised to investigate the influence of total race vibration exposure (VIB) on cell subset redistribution from pre- to post-one-hour time points (%Δcellspre-post1h) at the international race event while controlling for Lucia’s training impulse of the race event (LuTRIMP). Following multicollinearity checks to ensure Pearson’s bivariate correlation values between LuTRIMP and VIB were <0.8, a simple linear regression was performed (model1 = %Δcells_p_re-post1h~LuTRIMP) to control for LuTRIMP. Then, a second model was created to include VIB (model 2 = %Δcellspre-post1h~LuTRIMP + VIB) where standardised beta estimates (*β*) were generated [40] to calculate the contribution of each component to the overall predictive ability of model 2. The fit of the models was compared [41] and the ability of VIB to predict changes in %Δcellspre-post1h was considered as the change in *R*^2^ (Δ*R*^2^) between models [41]. Therefore, VIB was able to significantly predict variation in %Δcellspre-post1h when the change in *F-*ratio from model 1 to model 2 was significant (*p* < 0.05). Multiple linear regression tests were performed using R version 3.6.3. with packages car (version 3.0-2 [41]) and lm.beta (version 1.5-1 [40]).

## 3. Results

### 3.1. Overview

One participant failed to finish the race event due to a mechanical failure and was removed from subsequent analyses. Peripheral blood samples could not be obtained from the female participant at any time point and she was subsequently removed from all analyses. Stage 1 was removed from the vibration analyses as the GPS accelerometer units failed to pick up a signal for three participants. The remaining seven participants completed the race event successfully and all finishing in the top ten positions of the elite male category.

### 3.2. Laboratory Tests

The mean duration of the laboratory test was 27 ± 0.45 min and the group mean peak power output was 407 ± 15.5 W (5.4 ± 0.3 W/kg); the group mean power output at FBLC2 and FBLC4 was 280 ± 25 W (3.7 ± 0.2 W/kg) and 314 ± 18 W (4.1 ± 0.2 W/kg), respectively, with a corresponding HR of 160 ± 7 bpm (FBLC2) and 171 ± 5 bpm (FBLC4) highlighting the elite nature of the participants [25]. A significant increase in cell count post exercise as compared with pre- and post-1h values was shown for all cell subsets with the exception of CD4 senescent, CD4 effector memory, CD8 late, and CD4 late cell subsets (data not shown). An overview of cell subset redistribution including one way ANOVA results are presented in Table 2.

### 3.3. International Race Event

**Figure 3:**
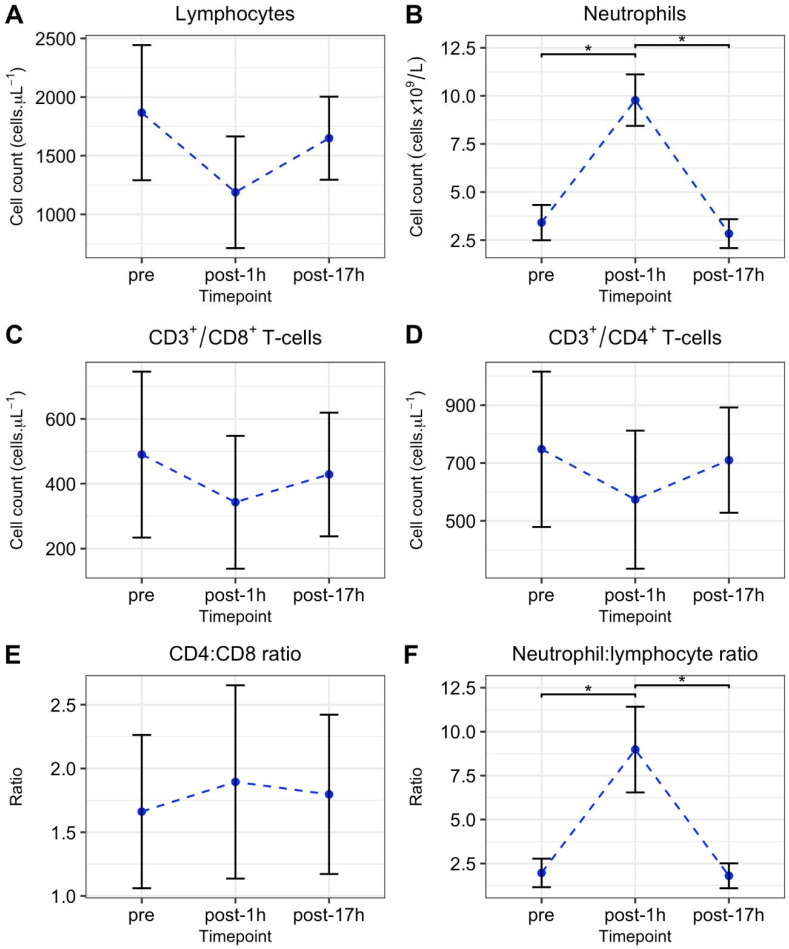
Mean ± SD (n = 7) total lymphocyte (**A**); neutrophil (**B**); CD8^+−^ T-cell (**C**); CD4^+^ T-cell (**D**) counts; and CD4:CD8 (**E**) and neutrophil:lymphocyte (**F**) ratio, before (pre), 1 h after (post-1h), and 17 h after (post-1h). * denotes significant difference between time points (*p* < 0.05, Bonferroni corrected) in the presence of a significant effect of time point (*p* < 0.05).

The mean LuTRIMP value for the total race event (race stages and transitions) was 370 ± 47 A.U. and the total duration was 5 h 30 min for all participants, as dictated by designated start and finish times. The mean race time (race stages only) was 1443 ± 24 s. A significant increase in neutrophil total cell count was observed from pre to post-1h time points before returning to pre values 17 h after the international enduro race (*F*(1.07,5.35) = 191.5, *p* < 0.001, ω^2^ = 0.90). As detailed in Figure 3, lymphocyte, CD8^+^ T-cell, and CD4^+^ T-cell total cell counts did not change significantly from pre-exercise values (*p* > 0.05).

**Figure 4:**
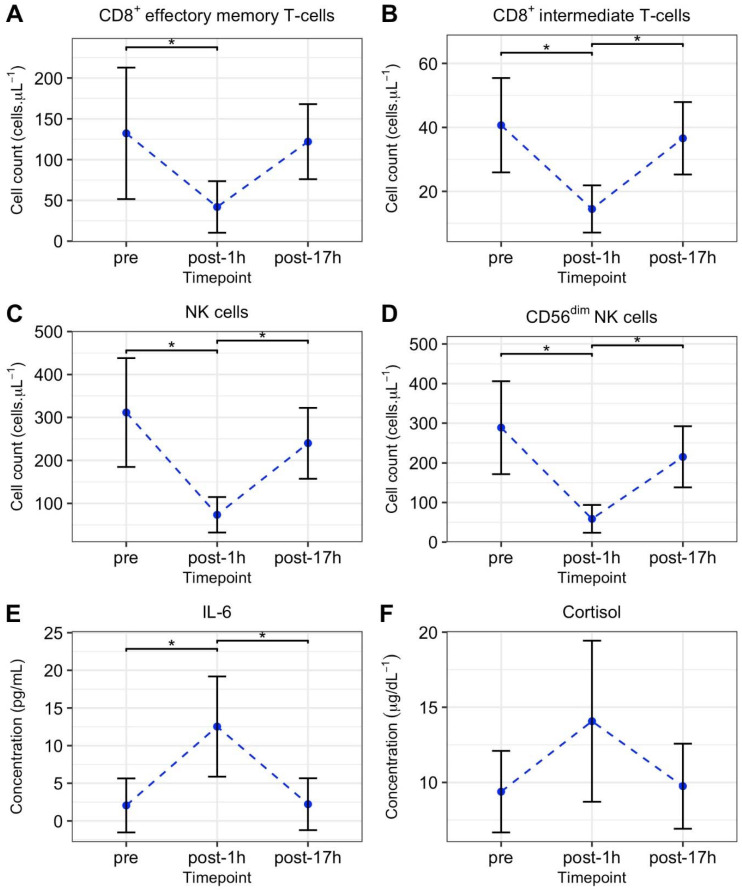
Mean ± SD CD8^+^ effector memory T-cells (**A**), CD8^+^ Intermediate T-cells (**B**), NK cells (**C**), CD56^dim^ NK cells (**D**) counts and IL-6 (**E**) and cortisol (**F**) concentrations before (pre), 1 h after (post-1h), and 17 h after (post-17h). A one-way ANOVA was used to investigate differences in blood sample data at each time point. * denotes significant difference between time points (*p* < 0.05, Bonferroni corrected) in the presence of a significant effect of time point (*p* < 0.05).

A significant reduction in CD8^+^ effector memory T-cell total cell count was observed from pre to post-1h time point (*F*(2,17) = 5.14, *p* = 0.018, ω^2^ = 0.29) as shown in Figure 4. The CD8^+^ intermediate T-cell total cell count was significantly reduced at the post-1h time point as compared with the pre or post-17 h time points (*F*(2,17) = 10.25, *p* = 0.001, ω^2^ = 0.48). Similarly, the total cell counts for NK cells and CD56^dim^ NK cells were significantly reduced 1 h after the race event as compared with the pre and post-17h measures (*F*(2,17) = 12.6, *p* < 0.001, ω^2^ = 0.54 and *F*(2,17) = 13.71, *p <* 0.001, ω^2^ = 0.56, respectively). The IL-6 concentration was significantly increased 1 h after the international race event as compared with the pre or post-17h values (*F*(2,17) = 10.40, *p* = 0.001 ω^2^ = 0.49). No significant changes in cortisol concentration were observed at any time points at the international race event. See Figure 4 for further detail of IL-6 and cortisol concentration.

### 3.4. Comparison of Laboratory Test and International Race Event

**Figure 5:**
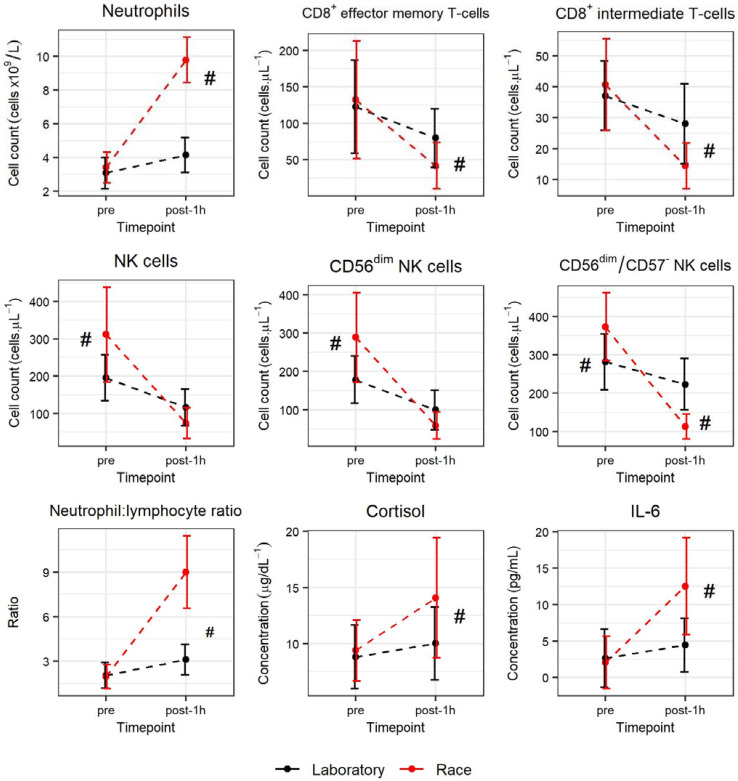
Mean ± SD total cell counts and concentrations (cortisol and IL-6) for laboratory test (‘Laboratory’) and international enduro race (‘race’). A two-way repeated measures ANOVA (test * time point) was used to investigate differences between tests (laboratory and race) and time points (pre and post-1h). # denotes ‘race’ significantly different from ‘laboratory’ in the presence of a significant interaction (test * time point), all *p* < 0.05 (Bonferroni corrected).

A significant interaction was observed for test * timepoint for neutrophil count (*F*(1,6) = 108.93, *p* = 0.000, ω^2^ = 0.94) where post hoc analysis revealed a significant increase following the race event as compared with the laboratory test, as shown in Figure 5. A significant interaction for test * timepoint was shown for CD8^+^ effector memory T-cell (*F*(1,6) = 25.11, *p* = 0.002, ω^2^ = 0.77) and CD8^+^ intermediate T-cell (*F*(1,6) = 9.78, *p* = 0.020, ω^2^ = 0.56) counts were significantly reduced 1 h following exercise (pre-post1h) following both the international race event and the laboratory exercise test. However, post hoc tests revealed this reduction was greater following the international race event as compared with the laboratory exercise test in both subsets, as shown in Figure 5. No significant interaction was observed for test * timepoint for the CD4/CD8 ratio. However, a significant interaction of test * timepoint was shown for the neutrophil/lymphocyte ratio (*F*(1,6) = 58.31, *p* = 0.000, ω^2^ = 0.89) with a significant increase in ratio after the race event as compared with the laboratory test (see Figure 5). A significant interaction for test * time point was discovered for CD56^+^ NK cell (*F*(1,6) = 59.54, *p* = 0.000, ω^2^ = 0.89) and CD56^dim^ NK cell (*F*(1,6) = 52.74, *p* = 0.000, ω^2^ = 0.88) total cell counts. Post hoc analysis showed significantly reduced counts for both subsets following both the international race event and the laboratory exercise test (see Figure 5). However, the total cell counts for CD56^+^ NK cells and CD56^dim^ NK cells were significantly higher before the international race event as compared with the laboratory-based exercise test (see Figure 5). A significant interaction (test * timepoint) was shown for the CD56^dim^/CD57^−^ NK cell total cell counts (*F*(1,6) = 48.35, *p* = 0.000, ω^2^ = 0.87) and post hoc analysis showed that the cell count was reduced 1 h after the international race event but not 1 h after the laboratory exercise test. The total cell counts for CD56^dim^/CD57^−^ NK cells were also significantly higher before and lower after the international race event as compared with the total cell count for the laboratory exercise test; see Figure 5 for further details. There was also a significant interaction (test * timepoint) for cortisol (*F*(1,6) = 6.76, *p* = 0.041, ω^2^ = 0.45) and IL-6 (*F*(1,6) = 24.04, *p* = 0.004, ω^2^ = 0.79) with concentrations significantly greater one hour after the race event as compared with those of the laboratory test, as shown in Figure 5.

### 3.5. Vibration, Training Impulse, and Redistribution of Lymphocyte Subpopulations

The mean total vibration exposure for the race event was 62.9 ± 3.4 ms^−2^ and the mean LuTRIMP for the total race was 369.7 ± 47.3 A.U. LuTRIMP and VIB were not significantly correlated (R = −0.5, *p ≥* 0.2) and simple linear regression showed no significant relationship between LuTRIMP and %Δcellspre-post1h for any cell subset. Relationships between %Δcellspre-post1h and VIB after controlling for LuTRIMP are shown in Table 3 below. A significant relationship was observed between vibration exposure and %Δcellspre-post1h for CD4^+^ T-cells, CD4^+^ early T-cells, CD4^+^ naïve T-cells, and NK cells.

## 4. Discussion

The purpose of this study was to investigate the redistribution of leukocyte cell subsets, IL-6, and cortisol in response to a laboratory-based maximal exercise test and at an international race event by elite enduro mountain bike athletes. The authors also aimed to investigate the relative contribution of training impulse and vibration on cell subset mobilisation 1 h after an international enduro mountain bike race. There was a significant redistribution of cytotoxic lymphocyte cell subsets of both the innate and adaptive components of the immune system one hour after the international race event before all cell counts returned to pre event values by the morning after the event. There did, however, appear to be a different immune response to the international race as compared with the laboratory-based maximal incremental testing, as %Δpre-post1h in cell subsets with potent effector functions was significantly greater at the race event. This suggests the race event offers a greater challenge to the immune system than a laboratory-based maximal exercise test. Whole body vibration was shown to be a superior predictor of %Δpre-post1h CD4+ T-cells, CD4+ early T-cell, CD4+ naïve T-cells, and NK cells as compared with LuTRIMP, suggesting whole body vibration is potentially a key component of training load measurement in enduro mountain bike applications.

The redistribution of leukocyte cell subsets after (post) and 1 h after (post-1h) laboratory exercise in the current study is similar to that presented previously, with the largest magnitude of ingress (pre-post) observed in CD8+ and NK cells [9]. The NK cells and cytotoxic T-cell subsets expressing potent effector functions are preferentially mobilised in response to psychological and physiological stressors via β2-adrenergic receptor stimulation [9,16]. The mobilisation of NK cells and cytotoxic T-cells is largely reliant on catecholamine signalling via the β2-adrenergic receptor [42], expression of which has previously been shown to reduce during periods of heavy training in moderately trained individuals [13].

The reduction in peripheral blood cytotoxic NK cell subsets and T-cell subsets 1 h after the international enduro mountain bike race may offer a protective or suppressive immune effect but the exact location during recovery from exercise is not currently known in humans. The CD8+ effector memory T-cells do not express the CD62L homing ligand, suggesting these cells are not returning to sites of likely infection such as the secondary lymphoid tissues [42]. The CD8+ intermediate and effector memory T-cells, and CD56dim NK cells exhibit potent cytotoxic functions as a common feature. Therefore, in the current study, these cells appear to be preferentially redistributed away from the peripheral blood during recovery from exercise due to their potent cytotoxic capacity, and for T-cells, depending on the retention of proliferative capacity. This recruitment away from the peripheral blood may serve to increase immunosurveillance in other tissues, however, the destinations of these cells in humans require further investigation [36]. Cells expressing CD3, including all T-cell subpopulations, have previously been shown to relocate to the Peyer’s patches, lungs, and bone marrow in response to acute exercise in mice [43]. This has been proposed to be a positive response that serves to increase immune vigilance at potential sites of infection following exercise [6,36]. Furthermore, no clinical link has been made between exercise-induced lymphopenia and risk of infection. Therefore, it is suggested that the reduction in cytotoxic lymphocyte cell subsets, as observed in the current study, is not detrimental to the athlete and may, instead, offer increased protection against infection during recovery from racing [6]. A substantial increase in neutrophil count one hour after the race is likely due to cortisol-induced release of neutrophil reservoirs from the bone marrow, however, values are slightly less than the increases observed following marathon running [22,24]. Increased circulating neutrophils is proposed to be a mechanism of protection against infection or injury associated with exercise, and further to repair damaged tissue during the recovery from exercise [44]. Neutrophils can also contribute to the activation, orientation, and expression of lymphocyte immune responses, including influencing NK and T-cell activity [45]. Thus, a transient increase, such as that observed here, is proposed to be beneficial to the host by means of increasing orchestration of immune response to potential challenges.

All cell subset counts were comparable to resting values on the morning following the event, demonstrating that one day of international competition has an acute effect on the redistribution of leukocyte cell subsets in elite enduro mountain bikers. These results are similar to data from hill and marathon runners [24,46] and are particularly interesting as the enduro athletes of the current study are often required to compete or train on multiple subsequent days. A return to baseline values of all cell subset counts is of benefit to the cellular immunity of the host, and therefore suggests these athletes are able to recover prior to subsequent days of competition. However, these findings must be interpreted with caution as the resting cell count does not reflect potential changes in cellular function [47] and subsequent response to exercise on the following day, which may differ as shown previously [46]. Accordingly, investigation of cell function and immune response to exercise on the subsequent day requires further research.

As the race event was of considerably longer duration as compared with the laboratory test, it is to be expected that the neutrophil response is significantly greater an hour after the race event [11]. Similarly, a decrease of CD8+ effector memory and intermediate T-cell subsets from post to post-1h is dependent on exercise duration and intensity, as reflected by a greater reduction at the race as compared with the laboratory test [9]. Interestingly, the reduction of the NK cell subsets from pre to post-1h shown here is also greater, but pre values are significantly increased prior to the race as compared with the same time point at the laboratory test. The NK cells comprise the leukocyte cell subset which is most responsive to psychological and physiological stress. As resting blood samples were taken prior to exercise, and thus in the absence of physiological stress, it is suggested that the psychological stress associated with the upcoming race may be the driver of increased resting counts of NK cells [16]. This has previously been suggested as a protective mechanism where psychological stress acts to ‘prime’ the immune system before the stressful event has even happened [16]. This priming has not been shown in an elite population prior to competition, however, it appears to be a healthy response to psychological stress prior to competition, as all athletes went on to finish in the top 10 in the elite category. In addition to alterations in leukocyte cell subset counts, IL-6 concentration was significantly increased 1 h after both the laboratory-based maximal exercise test and the international race event, in agreement with previous findings [11]. The IL-6 concentration is much greater following the race event which may serve as a means to increase delivery of energy substrate to working muscle via upregulation of hepatic glucose production and increase of fat oxidation [48]. Cortisol concentration showed a non-significant increase following the race event; however, the cortisol stress response is often highly individualised [49] and, as such, changes may not be reflected in group mean data presented here. Overall, a significant increase in neutrophil count, neutrophil/lymphocyte ratio, and IL-6 concentration alongside significant reductions in absolute count of cytotoxic cells shows that an inflammatory environment is created during the recovery from the race event [50].

While the demands of an enduro race event on the aerobic and anaerobic system have been documented previously [25], the immune response to mountain biking was not clear. The results of the current study show no significant relationship between traditional means of measuring training load (LuTRIMP) and leukocyte subset distribution one hour after the race. Conversely, greater vibration exposure increases the percentage reduction in CD4+ T-cells, CD4+ early T-cells, CD4+ naïve T-cells, and NK cells in the peripheral blood compartment one hour after the race event. This finding supports previous evidence showing the magnitude of CD4+ T-cell redistribution to be proportionate to the frequency of vibration exposure [27]. Our data further suggest that naïve and early CD4+ T-cell subsets are preferentially mobilised in response to vibration exposure, perhaps to relocate to secondary lymphoid tissue for potential activation, thus, increasing immunosurveillance, although further work is required in this area. A similar result was observed in NK cell populations, for the first time here, where the magnitude of egress of NK cells from the peripheral blood compartment is shown to be proportionate to the vibration exposure during the race event.

Although the underlying mechanisms remain unclear, these results show vibration to be a better predictor of leukocyte redistribution than the heart rate-based training impulse model (LuTRIMP). Interestingly, the LuTRIMP value of one day of enduro racing observed in the current study (370 ± 47 A.U.) is comparable to a flatter stage of the Tour de France (~350 A.U.) or Vuelta a España (~380 A.U.) [31]. It is, however, unlikely that these road cycling events also expose riders to the magnitude of vibration seen within individual stages here, which is more comparable to that observed during the cobbled sections of the Paris-Roubaix [51] or downhill mountain biking [52]. This suggests that, while enduro riders undergo substantial LuTRIMP assessed training volume, the concurrent vibration exposure is the key driver of the redistribution of leukocytes following enduro racing.

This has important implications for coaches and athletes aiming to optimise performance via careful prescription and monitoring of training load, often utilising training impulse models such as LuTRIMP. Instead, the results of the current study suggest that monitoring of vibration load in addition to training impulse in enduro athletes is recommended. Indeed, previous studies have shown that the addition of vibration to cycling exercise reduced the time to exhaustion [53] and increased the time at higher oxygen consumption but not higher heart rate [54], further demonstrating the importance of vibration in the measurement of training load in enduro mountain bike applications.

However, it should be noted that the current study is limited by vibration measurements which do not meet ISO regulations on whole body vibration [33] as appropriate equipment was not available at the time of data collection. Vibration measured at the seat post is also likely to differ from vibration measured at the handlebars [51,55] which may affect the results of the current study. Accordingly, thanks to advances in equipment, future studies should aim to investigate the relative contribution of vibration transmitted through the pedals, saddle, and handlebars in relation to the immune response of the athlete. Further studies are also recommended in female enduro athletes to assess potential sex differences in the immune response to enduro racing as immune response to muscle damaging exercise is blunted in premenopausal females as compared with males [56].

## 5. Conclusions

In conclusion, the international race event resulted in a more substantial transient relocation of cytotoxic cell subsets of both the innate and adaptive immune systems when compared to laboratory exercise values. While all values returned to resting levels by the following morning, the NK cell subset count was increased prior to international competition and appeared to prime the immune system for the race event, potentially as a result of psychological stress associated with competition. A significant relationship between total race vibration exposure and redistribution of CD4+ T-cells, CD4+ early T-cells, CD4+ naïve T-cells, and NK cells indicates vibration exposure is a possible driver of leukocyte redistribution in response to enduro mountain bike racing. Therefore, vibration should be taken into account when considering training load in mountain bike cycling disciplines.

## Figures and Tables

**Table 1:** Cell surface markers used for T-cell and NK cell subset identification.

Subset		Identification	References
T-cell (CD3^+^/CD4^+^ or CD3^+^/CD8^+^)	Naive	CD62L^+^/CD45RA^+^	[7,36]
	Central memory	CD62L^+^/CD45RA^−^	
	Effector memory	CD62L^−^/CD45RA^−^	
	EMRA	CD62L^−^/CD45RA^+^	
	Early	CD27^+^/CD28^+^	[37]
	Intermediate	CD27^+^/CD28^−^	
	Late	CD27^−^/CD28^−^	
NK cell (CD3^−^/CD56^+^)	Cytotoxic	CD56^dim^	[38,39]
	Regulatory	CD56^bright^	
	Early	CD56^+^/CD57^−^	
	Late	CD56^+^/CD57^+^	

**Table 2:** Overview of leukocyte circulating numbers of cell subset and cortisol concentration pre, post, and post-1h laboratory-based maximal exercise test (n = 7).

Variable	Time Point			Effect of Time Point
	Pre	Post	Post-1h	
Lymphocytes (cells·µL^−1^)	1581 ± 312	4432 ± 855 *	1388 ± 265 ^#^	*F*_1.44,6.62_ = 66.32, *p* < 0.001, ω^2^ = 0.86
Neutrophils (cells·µL^−1^)	3091 ± 921	5155 ± 1582 *	4164 ± 1035	*F*_2,18_ = 21.27, *p* < 0.001, ω^2^ = 0.28
CD8^+^ T-cells (cells·µL^−1^)	429 ± 142	1027 ± 329 *	350 ± 107 ^#^	*F*_1.06,6.39_ = 36.97, *p* < 0.001, ω^2^ = 0.65
CD4^+^ T-cells (cells·µL^−1^)	686 ± 177	1143 ± 219 *	637 ± 236 ^#^	*F*_2,18_ = 32.13, *p* < 0.001, ω^2^ = 0.51
NK cells (cells·µL^−1^)	196 ± 62	1605 ± 804 *	116 ± 49 ^#^	*F*_1.01, 6.06_ = 24.46, *p* = 0.002 ω^2^ = 0.67
CD4:CD8 ratio	1.7 ± 0.6	1.2 ± 0.5	1.9 ± 0.9	*F*_1.08,6.51_ = 23.44, *p* = 0.002, ω^2^ = 0.8
Neutrophil:lymphocyte ratio	2.1 ± 0.9	1.2 ± 0.5 *	3.1 ± 1.0 ^#^	*F*_2,18_ = 31.97, *p* < 0.001, ω^2^ = 0.43
Cortisol (µg·dL^−1^)	8.8 ± 2.8	10.2 ± 2.7	10.02 ± 3.2	*F*_2,18_ = 2.14, *p* = 0.16, ω^2^ = −0.05

* denotes significant difference from pre time point, ^#^ denotes significant difference from post time point (*p* < 0.05, Bonferroni corrected).

**Table 3:** Table of results of multiple linear regression controlling for LuTRIMP. Δ*F* = ΔF model1-model2, Δ*R*^2^ = Δ*R*^2^ model1-model2, *β*_LuTRIMP =_ standardised beta value for Lucia’s TRIMP, *β*_VIB_ = standardised beta value for vibration exposure. Model1 = %Δcellspre-post1h~LuTRIMP, model 2 = %Δcellspre-post1h~LuTRIMP + VIB.

Cell Subset	Δ*F_(_*_1,3)_	Δ*R*^2^	*p*	*β*_LuTRIMP_	*β*_VIB_
Neutrophils	0.05	0.01	0.835	−0.09	0.11
Lymphocytes	7.16	0.63	0.055	−0.30	−0.83
CD8^+^ T-cells	1.13	0.22	0.347	−0.09	−0.49
CD4^+^ T-cells	11.13	0.73	0.029 *	−0.18	−0.89
CD4^+^ Naïve (CD62L^+^/CD45RA^+^)	34.25	0.89	0.004 *	−0.25	−0.99
CD4^+^ Senescent (CD62L^−^/CD45RA^+^)	7.19	0.63	0.055	−0.07	−0.82
CD4^+^ early (CD27^+^/CD28^+)^	10.22	0.72	0.033 *	−0.21	−0.88
CD3^−^/CD56^+^ NK cells	4.12	0.63	0.050 *	−0.44	−0.83

* denotes significant change in F-ratio between model 1 (%Δcellspre-post1h~LuTRIMP) and model 2 (%Δcellspre-post1h~LuTRIMP + VIB).

## Data Availability

The data is contained within the manuscript.
